# Ratiometric imaging of calcium during ischemia-reperfusion injury in isolated mouse hearts using Fura-2

**DOI:** 10.1186/1475-925X-11-39

**Published:** 2012-07-19

**Authors:** Raghav Venkataraman, Mark R Holcomb, Rene Harder, Björn C Knollmann, Franz Baudenbacher

**Affiliations:** 1 Department of Biomedical Engineering, Vanderbilt University, Nashville, TN, USA; 2 Department of Physics, Susquehanna University, Selinsgrove, PA, USA; 3 Department of Electrical Engineering, Vanderbilt University, Nashville, TN, USA; 4 Departments of Medicine and Pharmacology, Vanderbilt School of Medicine, Vanderbilt University, Nashville, TN, USA; 5 Visiting Assistant Professor of Physics, Vanderbilt University, Nashville, TN, USA

**Keywords:** Optical Mapping, Langendorff Mouse Heart, Whole-Heart Fura-2, Ischemia-Reperfusion, Conduction Velocity, Reperfusion Injury, LAD ligation, Ratiometric Calcium Imaging

## Abstract

**Background:**

We present an easily implementable method for measuring Fura-2 fluorescence from isolated mouse hearts using a commercially available switching light source and CCD camera. After calibration, it provides a good estimate of intracellular [Ca^2+^] with both high spatial and temporal resolutions, permitting study of changes in dispersion of diastolic [Ca^2+^], Ca^2+^ transient dynamics, and conduction velocities in mouse hearts. In a proof-of-principle study, we imaged isolated Langendorff-perfused mouse hearts with reversible regional myocardial infarctions.

**Methods:**

Isolated mouse hearts were perfused in the Landendorff-mode and loaded with Fura-2. Hearts were then paced rapidly and subjected to 15 minutes of regional ischemia by ligation of the left anterior descending coronary artery, following which the ligation was removed to allow reperfusion for 15 minutes. Fura-2 fluorescence was recorded at regular intervals using a high-speed CCD camera. The two wavelengths of excitation light were interleaved at a rate of 1 KHz with a computer controlled switching light source to illuminate the heart.

**Results:**

Fura-2 produced consistent Ca^2+^ transients from different hearts. Ligating the coronary artery rapidly generated a well defined region with a dramatic rise in diastolic Ca^2+^ without a significant change in transient amplitude; Ca^2+^ handling normalized during reperfusion. Conduction velocity was reduced by around 50% during ischemia, and did not recover significantly when monitored for 15 minutes following reperfusion.

**Conclusions:**

Our method of imaging Fura-2 from isolated whole hearts is capable of detecting pathological changes in intracellular Ca^2+^ levels in cardiac tissue. The persistent change in the conduction velocities indicates that changes to tissue connectivity rather than altered intracellular Ca^2+^ handling may be underlying the electrical instabilities commonly seen in patients following a myocardial infarction.

## Background

Coronary heart disease affects approximately 1 in 8 adults and causes 1 in 6 deaths in the United States [1]. Occlusion of the coronary arteries can result in a myocardial infarction (MI) wherein affected cardiac tissue does not receive sufficient oxygenated blood. Ischemic cardiac muscle has reduced contractility and cannot pump blood efficiently; this condition can often lead to heart failure [2,3]. Patients are also likely to have lethal electrical disturbances such as ventricular tachycardia (VT) or fibrillation (VF) following ischemic attacks; half of the deaths occurring in a year following an MI are due to the sudden onset of disorganized electrical events [4].

A lack of oxygen during ischemia suppresses mitochondrial respiration leading to a low [ATP] state [5]. Low intracellular [ATP] slows the sarcolemmal Na^+^-K^+^-ATPase and the sarcoplasmic reticulum Ca^2+^-ATPase (SERCA) to directly change intracellular Ca^2+^ homeostasis and the homeostasis of other ions including Na^+^ and K^+^[6,7]. Slowing SERCA causes an increase in diastolic [Ca^2+^ as, with each beat, more and more Ca^2+^ is unloaded from the sarcoplasmic reticulum (SR). Simultaneously, at lower pH within ischemic tissue the Na^+^-H^+^-exchanger transports more Na^+^ into myocytes, forcing reverse-mode activity of the Na^+^-Ca^2+^-exchanger to facilitate a further increase in intracellular Ca^2+^[8].

This change in Ca^2+^ homeostasis has various effects as Ca^2+^ is arguably the most important ion in cardiac tissue [9]. Of interest to us, increased diastolic Ca^2+^ has been shown to act synergistically with lowered pH to suppress phosphorylation of connexins and close gap junctions [10]. This Ca^2+^ induced closure of the channel plays a vital role in a protective mechanism that isolates dying cells from healthy neighbors by protecting intact cells from depolarization [11]. However, this short term remodeling of gap junctions slows tissue activation and conduction velocity (CV) as gap junctions are the primary mechanism of cell-cell coupling [12]. Increased cytosolic Ca^2+^ concentration is hypothesized to lead to a higher propensity of ectopic beats in the MI border zone [13,14] and CV slowing increases the arrhythmogenic potential of myocardium, making the heart more susceptible to arrhythmias. Regional heterogeneities in CV can also facilitate wave break and form obstacles for waves to rotate about [15,16]. It is thought that damage caused by free radicals when the heart is re-oxygenated following an ischemic period make it more susceptible to electrical disturbances [17].

In efforts to study the physiology underlying this and other pathologies, Ca^2+^ has been imaged from whole-hearts by various groups primarily using the single wavelength dye Rhod-2 or the ratiometric dyes Indo-1 and Fura-2 [18,19]. The major benefit to using Rhod-2 is its high SNR, and that fluorescence from a heart’s surface can be excited by a green laser and recorded using a single high-speed camera. This allows the study of spatial changes in Ca^2+^ handling; with a sufficiently fast camera, CV can also be measured from such recordings [20]. Rhodamine based dyes have been used in our lab to quantify the changes in Ca^2+^ handling in myocytes and transgenic mouse hearts [21]. However, the single-wavelength nature of the dye necessitates the calculation of F/F_0_ and makes it susceptible to errors due to washout and photo-bleaching over the course of longer experiments [22,23]. As uneven loading occurs with all dyes, ascertaining spatial changes in calcium handling parameters such as diastolic Ca^2+^ and transient amplitude is difficult using a single-wavelength dye. Rhod-2 is used in dual-mapping studies, where transmembrane voltage and Ca^2+^ are imaged simultaneously using two cameras; in such experiments Rhod-2 can be used simultaneously with the voltage sensitive dye RH237 [19].

Notably, Indo-1 has been successfully used to image whole hearts using a custom-built lens assembly and two 256 photodiode arrays to record Indo-1 fluorescence with a temporal resolution of 1000 Hz [24]. The major drawback in using this dual-emission dye is the need for two detectors to obtain a ratiometric Ca^2+^ signal. When used in dual imaging studies with Di-4-ANEPPS, Indo-1 is typically used as a single-wavelength dye despite having two available detectors [24,25].

Fura-2 is attractive for whole-heart studies as the dye is dual-excitation with a single emission, and the ratio of fluorescence emitted provides a measure of absolute intracellular [Ca^2+^. If tissue parameters stay constant, Fura-2 fluorescence from multiple points on the heart’s surface can be directly compared, as can data across different hearts. The ratiometric nature of the dye makes it resistant to errors due to indicator leak or photo-bleaching. Fluorescence from fura-2 can be recorded on a single detector. Where multiple detectors are available Fura-2, whose spectra is comparable to Indo-1, could be used in conjunction with ANEPPS dyes or the pH sensitive BCECF [25-27] for multimodal imaging. Prior studies of intact hearts using Fura-2 have relied exclusively on photo multiplier tubes or fluorometers – generally coupled with optical fibers – to detect fluorescence [26,28-31]. In doing so fluorescence from a single small region of tissue is integrated and spatial information is difficult to discern. Furthermore, these studies switched illumination wavelengths at frequencies of 50 Hz to 250 Hz, in some cases entirely losing the ability to image transients.

In this study we present a simple quantitative method for imaging intracellular [Ca^2+^] from Langendorff-perfused mouse hearts with high spatial and temporal resolution using the ratiometric dye Fura-2. A switching light source is used to alternately excite the dye at two wavelengths and a CCD camera is synchronized to record the resulting fluorescence. Timing between the excitation source and CCD camera, critical for calculating ratios, is handled by a computer-controlled hardware timer. Our method allows monitoring of fluorescence from 1600 discreet spatial points while switching illumination wavelengths at an effective rate of 1000 Hz, significantly improving on current ratiometric imaging systems without using custom hardware. This spatiotemporal resolution is sufficient to determine the speed and patterns of conduction on the heart’s surface. If two cameras are available, the system described here could be easily modified to allow concurrent ratiometric Ca^2+^ measurements along with pH or transmembrane potential.

We imaged mouse hearts during ischemia and reperfusion to study the changes in intracellular Ca^2+^ concentrations and cycling dynamics, as well as conduction velocities. This serves as a proof-of-principle study and provides insights into the molecular mechanisms linking Ca^2+^ in ischemic episodes to changes in the conduction velocities and arrhythmias. With the immense potential of transgenic mice in studying various cardiac pathologies, this approach could be used widely to ratiometrically quantify Ca^2+^ and CV changes with a resolution not previously possible without specialized custom hardware [32].

## Methods

### Animal work

All animal work was performed in compliance with Vanderbilt IACUC. Bl-6 Mice were deeply anesthetized in a recumbent position with 5% isofluorane in oxygen. A thoracotomy was performed and the heart was rapidly excised and immersed in a cold bath of Tyrode’s solution containing heparin. The aorta was cannulated with a custom plastic cannula and knotted with silk suture to facilitate retrograde perfusion in Langendorff mode. A 5–0 silk suture (Ethicon Inc, NJ) was passed under the left anterior descending artery (LAD) and looped without applying pressure to the surface of the heart. The cannula was then connected to a constant-pressure perfusion system with Tyrode’s solution warmed to 37 °C and bubbled with 95% oxygen, 5% carbon dioxide. The Tyrode’s solution contained 130 mM NaCl, 4 mM KCl, 23 mM NaHCO_3_, 1.5 mM NaH_2_PO_4_, 1 mM MgCl_2_, 2 mM CaCl_2_ and 10 mM glucose (Fisher Scientific, PA).

### Dye preparation and loading

**Figure 1:**
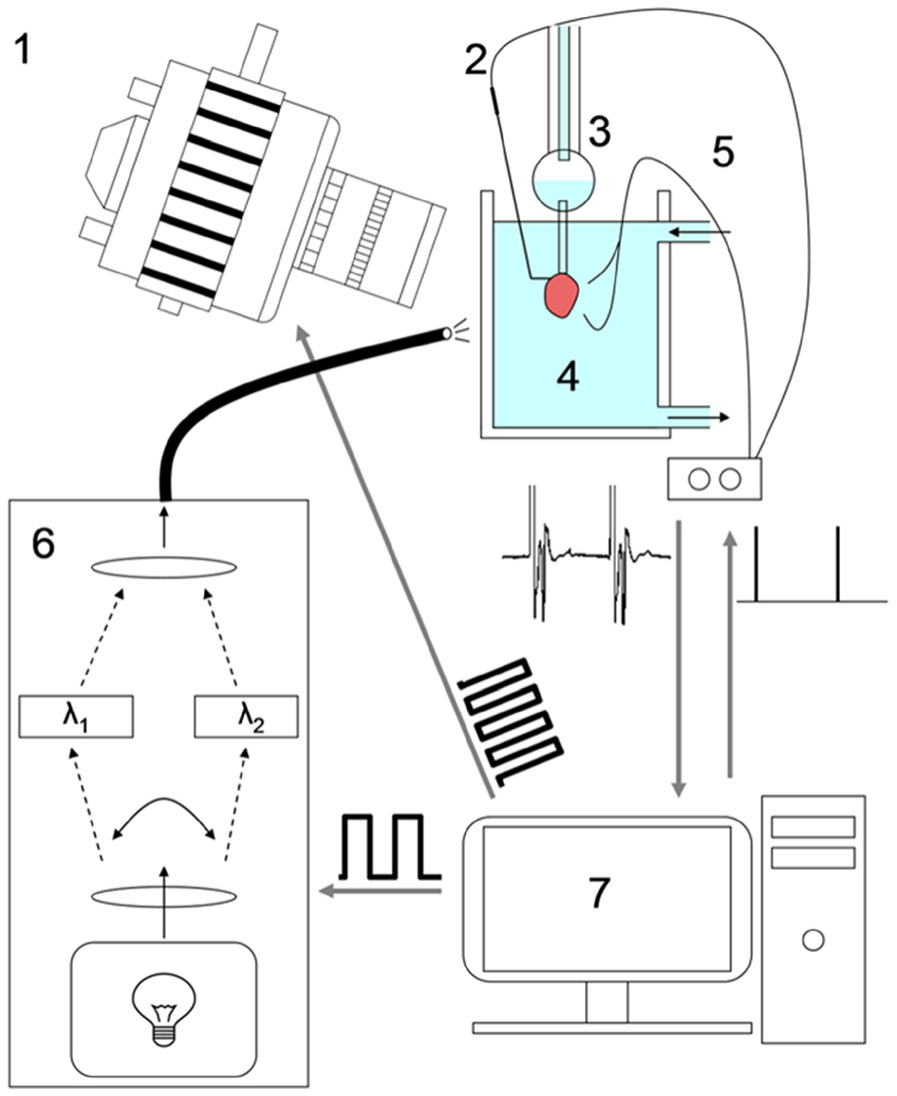
**Schematic drawing of experimental setup.** The mouse heart is perfused in Langendorff mode with warm oxygenated Tyrode’s solution delivered at a constant pressure through heat-jacketed tubing ( **3**). The heart is immersed in a re-circulated bath containing warm oxygenated Tyrode’s solution ( **4**) along with a cradle for two volume-conducted EKG electrodes and a platinum pacing electrode ( **2**). Illumination is provided at 380 and 340 nm by a high speed switching light source ( **6**). Emitted fluorescence passes through a 510 nm band-pass filter and is recorded on a high-speed CCD camera ( **1**). Timing for the illumination and the camera are provided by a custom computer program ( **7**); the pacing electrode and EKG leads ( **5**) interface with the computer through a PowerLab system.

25 μg Fura-2 AM (Invitrogen, CA) was dissolved in 20 μL of 5% Pluronic in DMSO (Invitrogen, CA) to produce a stock solution. This stock solution was diluted into 2 mL of Tyrode’s and sonicated for 10 minutes to produce a working solution. Working dye solution was placed in a small cup directly below the heart and re-circulated through the heart using a roller pump and a needle injected into the bubble trap (seen in Figure 1 above the heart). Following 15 minutes of recirculation, normal perfusion was resumed with the cup still in place for 10 minutes to allow the interstitial space to be washed free of unloaded dye. The cup was then removed, and the heart lowered into the heated bath.

### Fluorescence imaging system

The ratiometric dye Fura-2 AM requires illumination at two wavelengths, generally 340 nm and 380 nm. The ratio of fluorescence obtained at these wavelengths provides an estimate of intracellular [Ca^2+^]. In order to illuminate the heart with two wavelengths of light, a Lambda DG4+ light source (Sutter Instrument, CA) which allowed for high-speed switching between different optical filters was used. The DG4+ is capable of switching through 4 different excitation wavelengths at a rate of 1000 switches per second; we have utilized only two wavelengths at this rate in our study. The light source was fitted with optical excitation filters centered with pass-bands of 340 ± 15 nm and 380 ± 15 nm (Omega Optical, VT). The light source’s wavelength switching was driven by a computer generated TTL signal. The light source was also fitted with a third 460 ± 10 nm optical filter (Omega Optical, VT) to enable estimation of NADH autofluorescence, as described below.

The Fura-2 fluorescence spectrum is centered at 500 nm and can be imaged without interference from the illumination source using a band-bass filter in that range. A 40x40 pixel high-speed CCD camera (Redshirt Imaging, GA) equipped with a 510 ± 40 bandpass filter (Omega Optical, VT) was used for this purpose.

**Figure 2:**
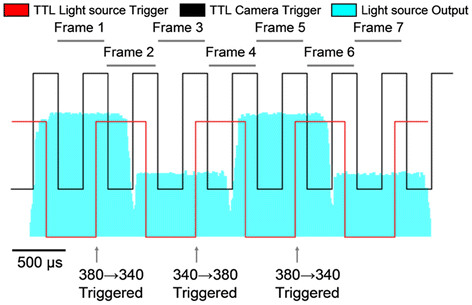
**Camera and light source timing*.*** The CCD camera is triggered by the falling edge of a 2KHz TTL signal (Black). The light source switching is triggered by the rising edge of a 1 KHz TTL signal (Red). Output from the light source was captured using a photo-diode, the output of which is shown in blue. The different amplitudes indicate the 2 different wavelengths of light supplied by the source. Odd numbered frames record fluorescence during stable illumination, while even numbered frames capture the transition period between the two wavelengths; these even numbered frames are deleted in post-processing.

The DG4+ light-source takes 0.5 ms to switch between adjacent filters. Selecting a switching frequency of 1 kHz allowed us to obtain stable light output for 0.5 out of every 1 ms. The camera was triggered twice as fast to record 2000 frames per second, with every other frame acquired when the light-source had reached stable output intensity at either 340 nm or 380 nm. This method also produced an equal number of unusable frames recorded during filter changes. Unusable frames were deleted during post-processing. Resulting data contained two interleaved recordings obtained at stable excitation intensities of either 340 nm or 380 nm, each at a sampling rate of 500 Hz. A timing diagram displaying the different signals as well as the light-source output intensity at both wavelengths is provided in Figure 2.

Acquisition of data and the generation of TTL timing signals were performed using a custom computer program interfacing a hardware timing board (National Instruments, TX). An empirically determined 0.4 ms delay was introduced between the camera and the light source triggers to compensate for the light source’s intrinsic switching lag.

### NADH autofluorescence correction

To account for the contribution of NADH to the measured Fura-2 fluorescence, we used the method described by Ylitalo et al. wherein flavin fluorescence at 460 nm provides an estimate of NADH autofluorescence [28]. Initially, we imaged dye-free hearts using excitation wavelengths of 340 ± 15 nm, 380 ± 15 nm to measure the NADH autofluorescence, and 460 ± 10 nm to measure flavin autofluorescence. NADH is in equilibrium with flavin and by plotting the fluorescence of one against the other, we produced curves to estimate the NADH autofluorescence at 340 nm and 380 nm from recordings taken at 460 nm.

During the course of our Fura-2 imaging experiments, recordings were taken at 460 nm immediately following the completion of each of our interleaved 340 nm/360 nm recordings. The contribution of NADH at 340 nm and 360 nm was then back-calculated offline from the recordings taken at 460 nm by using the previously generated calibration curves. Subtracting the resulting calculated autofluorescence values from the recorded fluorescence signal at 340 nm and 380 nm provided a measure of the true Fura-2 fluorescence.

### Experimental protocol

Following the dye loading protocol, the heart was rotated such that the left ventricle faced the camera and lowered into the heated bath. A small custom plastic cradle with 2 volume-conducting Ag/AgCl EKG electrodes was positioned around the heart with the electrode leads on either side of the heart. A platinum pacing electrode was positioned on the LV directly below the auricle of the heart. In this configuration, the cradle prevented the heart from swaying, while the electrode prevented the heart from rotating during contractions. A liquid light guide from the light source was connected to a bi-convex lens and aligned to allow full illumination of the area being imaged. Following 5 minutes in the bath, the heart was continuously paced throughout the experiment, except during fluorescence recordings when a pause was introduced, with a train of 2 ms wide S1 pulses supplied at a cycle length of 150 ms using a constant current source. The volume-conducted EKG was continuously recorded using a PowerLab (AD Instruments, CO) interface.

**Figure 3:**
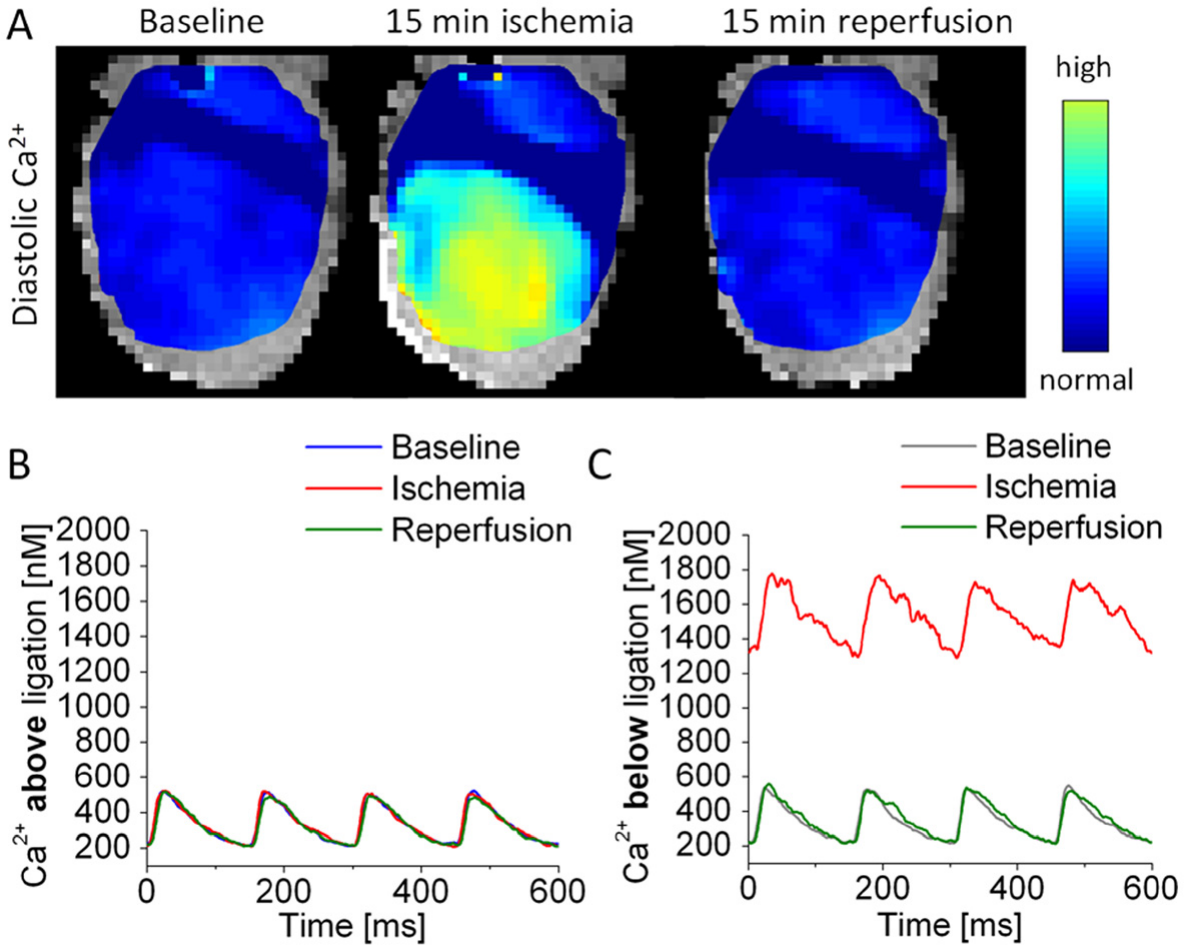
**Ligation of the left anterior descending artery produces a pronounced myocardial infarction.** Following cannulation of the aorta, a suture was inserted under the LAD and gently looped without applying pressure to the surface of the heart. Hearts were then loaded with Fura-2 AM and continuously paced at a cycle length of 150 ms near the base; Ca^2+^ transients were recorded from the surface of the left ventricle. (**A**, left) Initially, the diastolic Ca^2+^ is unchanged across the heart and calcium transients above and below the suture are identical. (**A**, middle) When the suture is tied off to create an MI, the diastolic Ca^2+^ rises dramatically in the ischemic region while staying normal in the region above the suture. (**A**, right) When the suture is untied and the MI is reperfused, diastolic calcium returns to normal as do the Ca^2+^ transients. (**B** and **C**) Transients from the well-perfused region above the suture stay consistent throughout the experiment, whereas ligation of the LAD caused significant reversible changes to diastolic Ca^2+^ and transient shape.

The heart was initially paced for 10 minutes without intervention to establish a steady baseline. The suture around the LAD was then tightened to create an MI in the lower section of the LV for 15 minutes. Finally, the suture was loosened to allow reperfusion into the ischemic region. Fura-2 fluorescence was recorded at 1–2 minute intervals throughout the entire protocol. During fluorescence recordings, four calcium transients initiated by S1 pulses were recorded; the pacing protocol was then changed to introduce a 500 ms pause to allow calculation of the time constant of transient decay. Steady state S1 pacing was resumed when the recording was complete. A representative section of a trace obtained from this protocol is provided in Figure 3A. This protocol was used to establish that the Fura-2 baseline fluorescence (R_B_) calculated at the end of the post-S1 pause was consistent across hearts, calculate the calcium transient decay time constant, and also to see if delayed after depolarizations leading to ectopic beats occurred during the post-S1 pause. Immediately following each interleaved 340/380 recording, we excited the hearts at 460 nm to estimate the contribution of NADH autofluorescence to the measured Fura-2 fluorescence.

Fura-2 can be calibrated using the formula [Ca^2+^ = k_d_·β·(R – R_min_)/(R_max_ – R), where k_d_ is the dye dissociation constant, R is the fluorescence ratio, β is F_380(min)_/F_380(max)_, and R_min_ and R_max_ are the minimal and maximal fluorescence ratios [26]. To ascertain R_max_ and F_380(max)_, hearts were infused with buffer containing 10 mM Ca^2+^, 10 μM of the Ca^2+^ channel agonist Bay K8644 (Sigma Aldrich, MO) and 10 μM of the SERCA blocker thapsigargin (Sigma Aldrich, MO); recordings were taken until steady Ca^2+^ fluorescence (R_max_) was obtained. To ascertain R_min_ and F_380(min)_, hearts were infused with buffer containing 5 mM EGTA (Sigma Aldrich, MO) and 0 Ca^2+^, and the fluorescence then recorded. This protocol could not be validated against established techniques as our attempts to use ionophores for the calibration failed; these efforts are detailed in the results section.

K_d_ for the dye in normal conditions and at various times during ischemia and during reperfusion was obtained using an equation relating pH and k_d_, along with previously published pH data for mouse hearts exposed to acute ischemia [26]. Normal k_d_ was calculated to be 143 nM, and k_d_ at 5, 10 and 15 minutes of ischemia was calculated to be 171, 237 and 374 nM respectively.

### Data analysis

Data analysis was performed using MATLAB (Mathworks, MA) and Origin (OriginLab, MA). The first step of post-processing involved removing frames recorded during light-source filter transitions. Following this, using the flavin fluorescence recordings and the pre-calculated NADH fluorescence calibration curves, the NADH autofluorescence contribution at excitations of 340 nm or 380 nm was calculated and subtracted from frames recorded with fluorescence excitation at the respective wavelengths. Finally, ratios of successive frames were taken to convert raw data into I_340_/I_380_ fluorescence ratios. This method resulted in ratios with a true frame rate of 500 FPS.

Maps of diastolic [Ca^2+^] were generated by determining the minima of the average of 4 transients taken from each point on the surface of the heart. These minimas were stored in an array to form a map of diastolic [Ca^2+^] over the heart.

**Figure 4:**
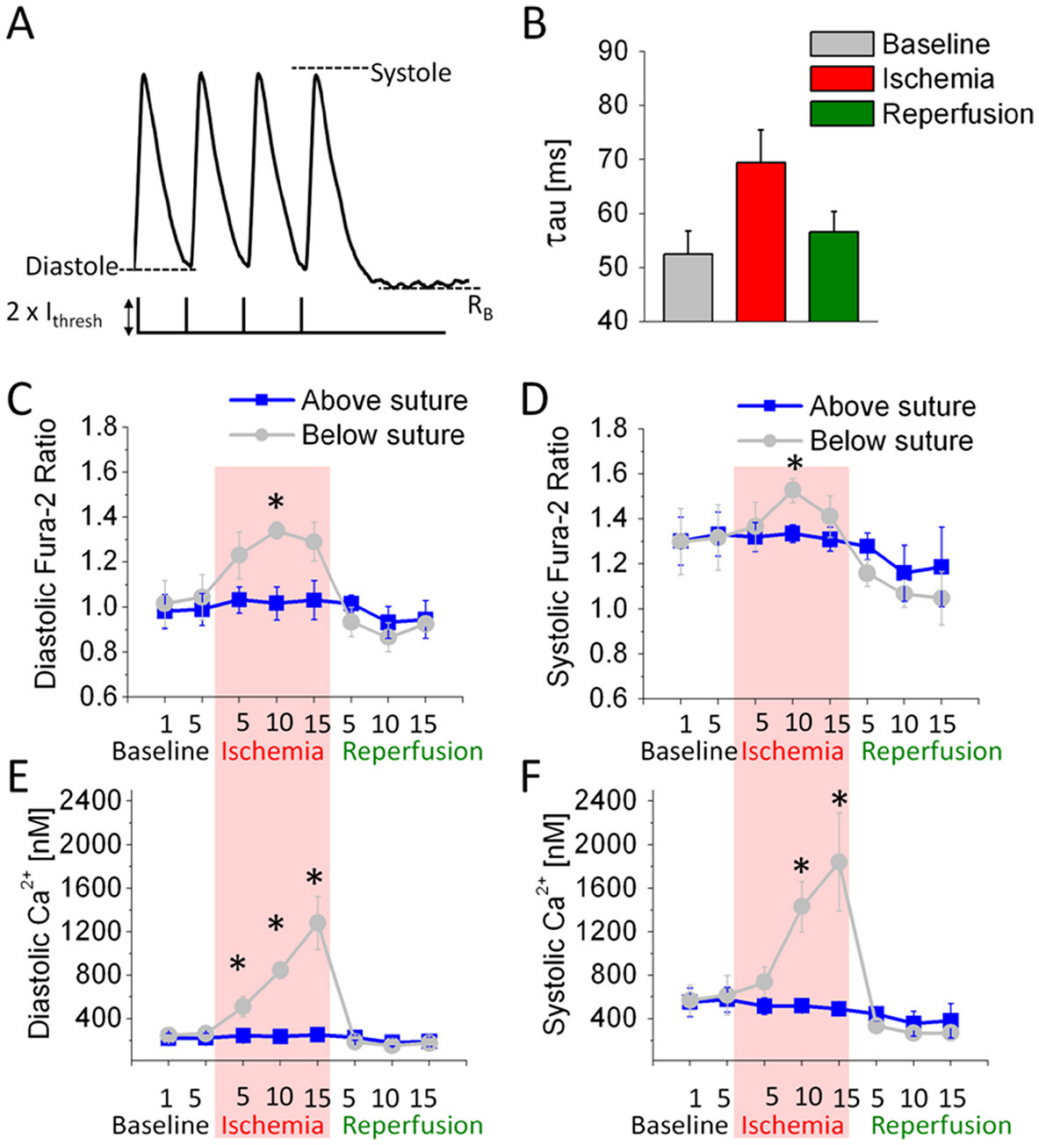
**Rapid reversible changes to Ca**^**2+**^**homeostasis occur during ischemia.** Hearts loaded with Fura-2 AM were subjected to a rapid pacing protocol consisting of a train of 2 ms wide S1 pulses at a cycle length of 150 ms. Pacing was continuously supplied through 5 minutes of normal heart activity, 15 minutes of ischemia and a further 15 minutes of reperfusion. Recordings of Ca^2+^ fluorescence and NADH autofluorescence were taken periodically throughout this time. During recordings alone, the pacing protocol was changed to introduce a 500 ms pause to allow calculation of the time constant of decay; when the recording was complete, steady state S1 pacing was resumed. Immediately following each Fura-2 recording, a second independent recording of flavin autofluorescence was made with an excitation wavelength of 460 nm to allow estimation of the contribution of NADH autofluorescence. (**A**) A representative recording of Ca^2+^ transients resulting from this protocol and the corresponsing stimulus pulses, each 2 ms wide at an amplitude twice the stimulation threshold (I_thresh_). (**B**) Single exponent fits of Ca^2+^ transient decays in the MI region indicate a slowing of the SR Ca^2+^ ATP-ase pump during ischemia which recovers during reperfusion. (**C**, **D**) NADH-corrected Fura-2 ratios indicate a moderate 40% increase in diastolic Ca^2+^ and 25% increase in systolic Ca^2+^ in the MI region. (**E**, **F**) Accounting for the pH-dependent changes in k_d_ that accompany ischemia shows the dramatic increase in both diastolic and systolic Ca^2+^ during ischemia in the MI region; both variables normalize during reperfusion.

To determine the conduction velocity (CV), videos were first filtered with a 3x3 spatial Gaussian filter and a 3 point moving-averaging temporal filter. An algorithm then automatically averaged 4 successive transients at each point on the surface of the heart and calculated the time of fastest rise, termed the activation time. These activation times were stored in an array to generate an activation map of the heart (Figure 4). Lines of interest were then manually selected along the direction of wave propagation, from base to apex, avoiding the ligation suture. Linear regression of the activation times and the distances along the line provided the CV along the line.

Ca^2+^ transients from a user-selected point on the LV were filtered with a 3x3 spatial Gaussian filter and a 3 point moving-averaging temporal filter. Filtered transients were exported into Origin (OriginLab, MA) and analyzed to manually determine various parameters (Figure 3). The Ca^2+^ transient decay rate was calculated by iteratively fitting the last decay of the last S1 transient to a single-exponential decay function in Origin.

Data was tested for significance using a two-sample two-tailed Student’s *T*-test assuming unequal variances in Excel (Microsoft, WA). Asterisks on figures of Ca^2+^ concentrations and transient amplitudes indicate a significant difference (p < 0.05) between the region above and below the suture for that metric, at that point in time. In figures of transient decay time (Tau) or CV, the asterisks indicate that within the region below the suture, there was a significant difference (p < 0.05) between values recorded at baseline and those recorded during ischemia.

## Results and discussion

### Fura-2 can be effectively used in whole mouse hearts

The acetoxymethylester (AM) form of Fura-2 can be loaded into Langendorff-perfused mouse hearts using a 25 minute loading protocol. Whereas correcting for the heterogeneous uptake of dye in the heart is difficult when using single-wavelength [Ca^2+^] dyes, taking the ratio of Fura-2 fluorescence at each pixel produces a homogeneous emission across the surface of the heart. This corrects the misleading heterogeneity in fluorescence seen while using single-wavelength dyes and permits the study of pathological spatial heterogeneities in mouse hearts. Furthermore, whereas leakage and photobleaching of single-wavelength dyes can cause temporal changes to emission intensity, the ratio of Fura-2 emission stays constant regardless of intracellular concentration.

During normal perfusion, Fura-2 ratio transients from different hearts can be compared directly against each other without the need to calculate a ratio to baseline fluorescence (F/F_0_), as in the case of single-wavelength dyes. R_b_ was similar in all hearts. In studies where tissue parameters do not change, it is not essential to calibrate the dye before making comparisons. During ischemia however, intracellular pH and thus the dye’s k_d_ change significantly, necessitating calibration to compare normal transients to ischemic ones. We initially attempted to calibrate the Fura-2 fluorescence using an ionophore to set the intracellular [Ca^2+^ as reported by others [26]. Ionomycin, Triton-X100 and 4Br-A23187 were tested at various concentrations under different loading conditions, but none of the three gave significant or reproducible changes in fluorescence. Previous studies have had varying success in calibrating Ca^2+^ fluorophores – whereas some have been able to calibrate Fura-2 and Rhod-2, others have reported difficulties while using ionophores [23,26,33-35]. Instead, we used the L-type Ca^2+^-channel opener Bay-k 8644 in combination with the SERCA blocker Thapsigargin in a high-Ca^2+^ setting to increase the Fura-2 fluorescence ratio and obtain an R_max_ dramatically higher than the peak systolic Ca^2+^ in ischemic tissue. A heavily buffered 0 Ca^2+^ solution was perfused through the heart used to obtain R_min_.

We found that due to the dual-wavelength excitation system, changing the orientation of the excitation light guide changed the resulting emission ratio significantly. This importance of the angle of illumination could result from differential absorbance and diffraction of the two excitation wavelengths. It is therefore important to maintain the illumination geometry of the imaging setup.

### LAD ligation creates a well defined myocardial infarction (MI)

**Figure 5:**
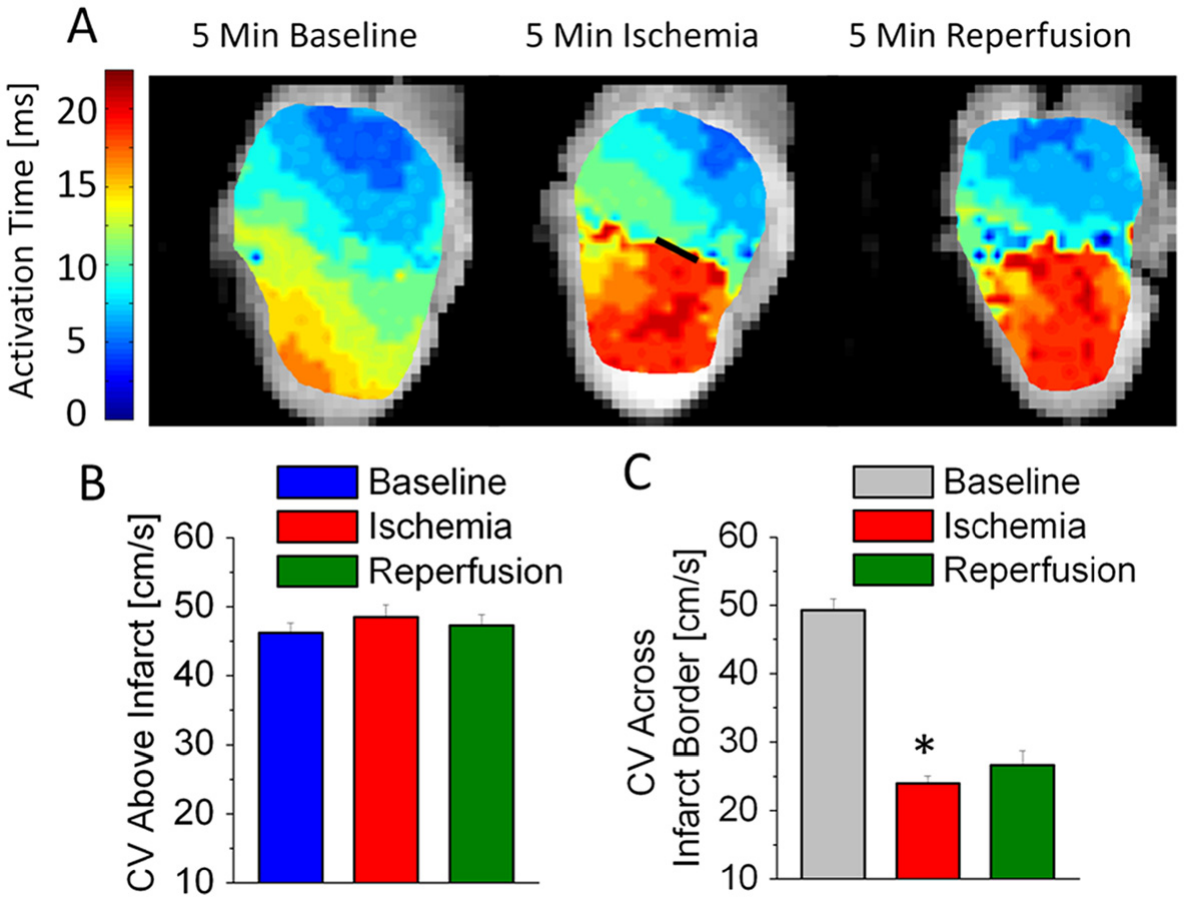
**Conduction velocity on the ventricular surface slows dramatically across the infarction border and does not recover.** Recordings of Ca^2+^ fluorescence were analyzed offline to determine the CV of the activation wavefront on the ventricular surface. (**A**) Plotting a map of activation times along the surface of the heart indicates that tissue in the MI region activates much later than normal during both ischemia and reperfusion. The black line in the ischemia map indicates the region of suture constriction ( **B**) CV in the region above the suture is unchanged throughout the protocol. ( **C**) The CV across the border drops significantly by 5 minutes of ischemia and doesn’t recover during reperfusion (also shown at 5 minutes), reflecting the changes in activation times. N = 7-8 per group. * p < 0.05.

The left anterior descending coronary artery (LAD) is reproducibly ligated using a hooked surgical needle and fine suture. Passing 5–0 suture under the LAD and the gently looping it on the surface does not itself affect perfusion or damage tissue (Figures 5A left, 4B and D). Tightening the suture produces a very well defined infarct in the LV; this can be visualized by mapping the rise in diastolic [Ca^2+^] below the suture (Figure 5A middle). The rise in fluorescence is given in arbitrary units as the pH gradient across the border is not known. The MI has a sharp, well defined border with uncalibrated Fura-2 fluorescence ratio quadrupling across the border over a distance of 0.6 mm. This method provides a control and ischemic region in each heart as the area above the suture is not affected (Figure 5B), while the area below is infracted (Figure 5C). A thinner suture would obscure less of the heart’s surface, but our attempts to use 9–0 suture caused the LAD to tear.

### Ischemia induces reversible changes in Ca^2+^ handling

The lower pH and lack of ATP caused by ischemia affect various ATPases and ion channels causing decreased contractility and Ca^2+^ accumulation in the cytosol. Furthermore, the rising proton concentration [H^+^ within the cell leads the Na^+^-H^+^-exchanger to transport Na^+^ into myocytes. Increasing intracellular Na^+^ forces reverse-mode activity of the Na^+^-Ca^2+^-exchanger to facilitate a further increase in intracellular Ca^2+^[8].

The change in the fluorescence ratio of diastolic [Ca^2+^ is rapid and reaches a maximal level in within 10 minutes of the start of regional ischemia (Figure 3C). However, the dye’s dissociation constant is extremely pH-dependent and the estimated diastolic Ca^2+^ continues to rise throughout the ischemic period with falling pH (Figure 3E) [22,26,28,36]. We used k_d_ values calculated in a previous study where hearts were subjected to global ischemia while being imaged inside an NMR magnet [26]. Normal k_d_ was calculated to be 143 nM, and k_d_s at 5, 10 and 15 minutes of ischemia were calculated to be 171, 237 and 374 nM respectively. From our measurements we estimated that at baseline the average diastolic [Ca^2+^ was 223 nM, the systolic [Ca^2+^ 553 nM and therefore the transient amplitude 330 nM (Figure 3E and F). Our values are higher than those previously published from Fura-2 loaded hearts [26] and lower than values seen in hearts imaged with Rhod-2 [18,37,38]. This deviation is not surprising since calibration, dye loading, pacing frequency and history, temperature, perfusate [Ca^2+^ and perfusion pressure, among other factors, affect Ca^2+^ measurements. Furthermore, in our efforts to calibrate the dye’s fluorescence, we have implicitly assumed that progression of ischemia and the accompanying changes to cellular Ca^2+^ handing are identical to the cited study from which k_d_ was obtained; minor variations would affect the absolute number estimated by us.

During 15 minutes of ischemia the diastolic Ca^2+^ climbs to more than five times its baseline value. Over the period of acute ischemia the Ca^2+^ transient amplitudes does not change significantly; a non-significant increase in transient amplitude may be due to decreased cytosolic Ca^2+^ buffering by the pH-mediated myofilament desensitization. Similar results have been seen in isolated myocytes exposed to comparable periods of ischemia [39], and in intact hearts exposed to shorter [40,41] and comparable periods of ischemia [42]. Other reports have shown increases and decreases in Ca^2+^ transient amplitudes during acidosis [43-45]. Contrasting the calibrated estimates, the uncalibrated Fura-2 transient amplitude seen in our experiments decreases dramatically during ischemia, further highlighting the need for fluorophore calibration when the local pH changes.

SERCA normally operates close to its free energy equilibrium and changing [ATP] is known to affect its ability to pump against a concentration gradient [6]. Fitting the last S1 Ca^2+^ transient decay to a single exponential decay curve confirmed that SERCA was slowed as the Ca^2+^ transient decay time constant increased dramatically (Figure 3C) during ischemia. In ischemic tissue the 69 ms time constant was significantly longer than the 52 ms or the 57 ms seen in normal and reperfused tissue respectively (p < 0.05); normal and reperfused tissue were not significantly different from each other. During acute ischemia as the SERCA slows and diastolic Ca^2+^ increases, it is likely that the SR Ca^2+^ store is slowly depleted by maintaining constant Ca^2+^ transients. Over long periods the SR-Ca^2+^ store is expected to deplete, affecting the transient amplitude.

We did not observe evidence of ectopic activity in any of the hearts when the S1 train was purposely paused for 500 ms during recordings. Spontaneous Ca^2+^ release from the SR in the MI border zone is hypothesized to occur due to an increased SR load in cells in close proximity to Ca^2+^ overloaded regions due to diffusion across gap junctions [46]. However, the observed fast diastolic Ca^2+^ rise leading to cell-cell uncoupling would counteract this proposed mechanism for ectopic activity. Spontaneous RyR2 Ca^2+^ release events due to the increased diastolic Ca^2+^ might also be compensated by depressed RyR2 gating at low pH_i_[47,48].

During reperfusion Ca^2+^ handling in the MI region recovers to baseline values but a non-significant decrease in diastolic and systolic Ca^2+^ is visible (Figure 3B and D). This trend is immediately evident in the infarcted area, but also develops in the healthy region above the suture. This may be a secondary effect of occluding the coronary artery to induce reperfusion injury.

A third of the Fura-2 AM loaded into myocytes is compartmentalized by cellular organelle including the mitochondria [49,50]. In our experiments, we did not correct for the fluorescence caused by different compartments. It is therefore possible that mitochondria, which form 20-40% of cardiac tissue, contribute to the fluorescence recorded [51]. The large increase in diastolic [Ca^2+^ seen during ischemia may partially be influenced by mitochondrial Ca^2+^ buffering [52]. This mitochondrial fluorescence affects other Ca^2+^ indicators as well, and potentially leads to an overestimation of the diastolic cytosolic Ca^2+^ concentration but does not affect CV calculations or the overall findings of this study.

### Ischemia induces irreversible changes in CV

Insertion of the suture under the LAD does not initially obstruct conduction along the surface of the heart (Figure 4A left). When the suture is tied off, the infarcted region alone takes dramatically longer to activate, implying a slowing in CV (Figure 4A center) across the border zone. Higher [Ca^2+^ (Figure 5 center) has been demonstrated to affect phosphorylation of connexins in gap junctions – the primary determinant of cell-cell communication, ion transfer and conduction velocity [10].

In reperfusion, activation of the MI region remains delayed after diastolic [Ca^2+^ returns to normal. This is likely due to permanent damage caused to the intercalated discs during the ischemic period [53]. It is possible that changes to various tissue parameters, including pH, that also affect gap junction conductance do not normalize rapidly during reperfusion [10]. Supporting our findings in the whole heart, a study of rat ventricular myocyte monolayers exposed to ischemia found that [Ca^2+^ normalizes rapidly in reperfusion, but CV recovery is delayed [54]. An earlier study of dog hearts similarly found that CV continues to be suppressed by > 50% in reperfusion, following brief periods of ischemia [55].

It is likely that the suture does some damage to a small strip of the epicardium when tightened (region under suture loop shown as a black line in Figure 4A center). To avoid the potential confounding effects of this damage, CV was not measured over or near the suture, but rather on either side of the suture.

The process of CV calculation does not take into consideration the fiber orientations and therefore does not allow us to determine the anisotropy ratio of the CV. We were unable to consistently calculate the CV inside the MI region due to the small surface area of the mouse heart and possibly because activation sometimes occurs transmurally rather than epicardially inside the MI. It is also important to consider that the studies of CV generally employ transmembrane voltage (Vm) mapping rather than Ca^2+^[56]. Our method of calculating CV from Ca^2+^ relies on consistent coupling between Ca^2+^ and Vm, and it is possible that changes in this coupling occur during ischemia as the Ca^2+^ transient shape changes. However, since the Ca^2+^ transient shape normalizes during reperfusion while the CV remains suppressed as it was during ischemia, we do not believe that our calculations are significantly biased by changes in the transient shape. Furthermore, as with Ca^2+^ transients, AP shape, amplitude and rise time are also affected by brief periods of ischemia [55].

## Conclusions

We have developed a method for imaging spatial and temporal changes in [Ca^2+^ from isolated mouse hearts using the ratiometric dye Fura-2. Using this ratiometric dye offers some benefits over traditional single-wavelength dyes including the ability to measure heterogeneity in diastolic [Ca^2+^ and independence from dye photobleaching and leakage over time. Furthermore, unlike Indo-1, this dye can be imaged using a single detector. This method significantly improves on the spatiotemporal resolution currently available for Fura-2 imaging. It has a wide range of potential applications as changes to [Ca^2+^ handling play a key role in various conditions including ischemia, heart failure and genetic heart diseases. Since this technique uses a generic off-the-shelf light source coupled with a CCD camera, it can be reproduced easily by other labs. With little alteration this method could also be applied to image other ratiometric dyes such as the transmembrane potential dye Di-4-ANEPPS [57].

In this study we have imaged the spatial extent and effects of ischemia-reperfusion injury in hearts with LAD ligations. Ligation of the LAD, similar to a heart attack, produces a region of ischemic myocardium. Ischemia causes a rapid significant rise in diastolic Ca^2+^ while dramatically slowing CV. Reperfusion corrects Ca^2+^ handling to normal but tissue CV remains significantly slowed. Slowing of CV during ischemia is correlated to a rise in [Ca^2+^], but the damage caused cannot be reversed by simply lowering [Ca^2+^]. Prolonged slowing of CV would render the myocardium pro-arrhythmic and potentially contributes the increased incidence of arrhythmias seen in patients following heart attacks. However, as we have demonstrated here, correcting for the changing k_d_ – necessary in ischemic preparations – is difficult in intact tissue. We believe that our method would be best utilized in settings where tissue parameters to not change, and the Fura-2 ratios can be directly compared.

Our results indicate that electrical instabilities immediately following ischemia are likely driven by changes in conduction velocities rather than by changes to intracellular Ca^2+^ cycling. Secondary effects may involve both changes to tissue properties and Ca^2+^ cycling. Based on the trends seen in our experiments (Figure 3), we predict that an extended experimental protocol would likely show long-term changes in Ca^2+^ handling.

## Abbreviations

AM: Acetoxymethyl ester; CV: Conduction Velocity; F/F0: Ratio of recorded fluorescence to baseline fluorescence for single wavelength dyes; FPS: Frames per Second; kd, Dissociation constant; LAD: Left Anterior Descending Artery; MI: Myocardial Infarction; RB: Baseline fluorescence ratio (I340/I380) for Fura-2; SERCA: Sarcoplasmic Reticulum Ca2+-ATPase; SR: Sarcoplasmic Reticulum; Vm: Transmembrane voltage.

## Competing interests

The authors declare that they have no competing interests.

## Authors’ contributions

RV: Participated in designing the study. Performed surgeries and experiments. Developed data analysis techniques and analyzed data. Made figures and drafted the manuscript. MH: Implemented the timing scheme used to synchronize camera and light source. RH: Developed hardware to measure light source-camera sync and participated in editing the manuscript. BCK: Contributed to the protocol to calibrate the ratiometric dye Fura-2. FB: Conceived of and designed the study. Developed data analysis techniques. Participated in drafting the manuscript and final edits. All authors read and approved the final manuscript.
